# Dual Activation of GLP-1 and AMPK Pathways by a Multi-Botanical Formulation Improves Obesity and Metabolic Dysfunction in Experimental Models

**DOI:** 10.3390/nu18132111

**Published:** 2026-06-28

**Authors:** Anna Goc, Waldemar Sumera, Aleksandra Niedzwiecki

**Affiliations:** Dr. Rath Research Institute BV, 5941 Optical Ct., San Jose, CA 95138, USA; w.sumera@drrath.com

**Keywords:** obesity, metabolic dysfunction, GLP-1, AMPK activation, nutritional intervention, phytochemicals, adipogenesis, inflammation, energy metabolism

## Abstract

Background: Obesity is a multifactorial metabolic disorder characterized by excessive adiposity, chronic low-grade inflammation, and dysregulated incretin and energy-sensing pathways, including glucagon-like peptide-1 (GLP-1) and AMP-activated protein kinase (AMPK). Methods: This *in vitro* and *in vivo* study evaluated the potential of select phytochemical candidates and botanical formulations to stimulate GLP-1 secretion and activate AMPK signaling. Results: Fourteen phytochemicals and six combinations were screened in human NCI-H716 enteroendocrine cells at 10–20 µg/mL to assess cytotoxicity and GLP-1 secretion. In human adipocytes, selected combinations reduced lipid accumulation and monocyte chemoattractant protein-1 (MCP-1) secretion. Among the tested formulations, combination #4, consisting of ginseng root extract, curcumin, white kidney bean extract, fenugreek extract, capsaicin, and bitter melon extract, significantly increased phosphorylated AMPK levels *in vitro*. In high-fat diet-induced obese mice, oral administration of combination 4 reduced body weight gain and white adipose tissue mass, improved metabolic biochemical parameters, restored leptin and MCP-1 levels toward normal values, increased GLP-1 level, and normalized GLP-1 receptor expression in subcutaneous adipose tissue. Conclusions: These preclinical findings demonstrate that this multi-component botanical formulation modulates GLP-1 secretion, AMPK phosphorylation, lipid accumulation, and inflammatory markers in cellular and murine models. These data provide a foundational rationale for its further evaluation as a non-toxic candidate for metabolic management.

## 1. Introduction

Obesity is a chronic, relapsing, and multifactorial disease characterized by excessive accumulation of adipose tissue that impairs health, increases morbidity, and shortens life expectancy [1]. In the United States, obesity affected approximately 40% of adults between 2021 and 2023, with severe obesity approaching 10% [2]. Globally, the World Health Organization estimated in 2022 that more than one billion people were living with obesity, representing more than twice the prevalence observed in 1990 [3]. Clinical management of obesity currently relies on sustained energy restriction, improved diet quality, and regular physical activity, often supported by pharmacotherapy [4]. Incretin-based therapies, particularly glucagon-like peptide-1 (GLP-1) receptor agonists, achieve clinically meaningful and sustained weight loss while improving glycemic control when combined with lifestyle modification [5]. For severe or refractory obesity, metabolic and bariatric surgery, especially sleeve gastrectomy and Roux-en-Y gastric bypass, remains the most effective intervention, producing substantial and durable weight reduction, high rates of type 2 diabetes remission, and reduced obesity-associated mortality [4].

The pathophysiology of obesity results from complex interactions among genetic, neuroendocrine, environmental, behavioral, and social factors [4,6,7,8]. At the neuroendocrine level, hypothalamic circuits integrate peripheral metabolic signals such as leptin, insulin, gremlin, GLP-1, and peptide YY to regulate appetite, satiety, and energy expenditure. In obesity, however, these pathways frequently exhibit resistance to anorexigenic signals, promoting positive energy balance and weight gain [1,4,8]. Obesity is also associated with impaired AMP-activated protein kinase (AMPK) signaling, a central regulator of cellular energy homeostasis that suppresses lipogenesis and promotes fatty acid oxidation [9]. These alterations contribute to adipocyte hypertrophy and hyperplasia, expansion of white adipose tissue (WAT), tissue hypoxia, and chronic low-grade inflammation characterized by increased production of pro-inflammatory cytokines, including MCP-1, TNFα, and IL-6, together with an adverse adipokine profile [10,11]. Excessive energy intake further promotes de novo lipogenesis and adipogenesis while suppressing lipolysis, thereby amplifying triglyceride storage [6]. Consequently, WAT acts not only as an energy reservoir but also as an active endocrine and inflammatory organ contributing to systemic insulin resistance, dyslipidemia, and vascular dysfunction [1,4,10,12].

At the nexus of systemic metabolic regulation lies a highly interconnected molecular network governed by the mTOR/SIRT1/PGC-1α signaling axis, which serves as a master rheostat for cellular energy homeostasis and mitochondrial function [13]. While metabolic midpoints like glucagon-like peptide-1 (GLP-1) and adenosine monophosphate-activated protein kinase (AMPK) are well-established drivers of metabolic health, their downstream phenotypic effects are tightly coordinated by this intricate energetic cascade [14]. Specifically, the activation of AMPK directly promotes the upregulation of sirtuin 1 (SIRT1) and the concomitant inhibition of the mechanistic target of rapamycin (mTOR), a crucial transition that shifts cellular machinery away from energy-consuming anabolic processes toward survival and efficiency [13,14,15]. Crucially, SIRT1-mediated deacetylation activates peroxisome proliferator-activated receptor-gamma coactivator 1-alpha (PGC-1α), the principal orchestrator of mitochondrial biogenesis, fatty acid oxidation, and respiratory chain efficiency [16]. Dysregulation of this precise signaling architecture is a foundational hallmark of diet-induced obesity and metabolic syndrome [17]. Consequently, pharmacological or botanical interventions capable of modulating the crosstalk within the mTOR/SIRT1/PGC-1α axis present a highly promising therapeutic strategy to restore metabolic equilibrium and mitigate aberrant weight gain [18].

Although GLP-1 receptor agonists and related therapies have transformed obesity treatment, they are associated with dose-limiting adverse effects, primarily gastrointestinal symptoms such as nausea, vomiting, diarrhea, constipation, abdominal pain, and dyspepsia, as well as transient dizziness, headache, mild tachycardia, and injection site reactions [19,20]. These limitations have intensified interest in naturally derived bioactive compounds, which are generally perceived as safer yet often demonstrate only modest efficacy compared with approved anti-obesity medications [21]. Major classes of natural anti-obesity compounds include polyphenols, alkaloids, terpenoids and carotenoids, many of which regulate pathways relevant to adipogenesis, inflammation, and energy metabolism [16,17,18,19,20]. Polyphenols such as quercetin, EGCG, resveratrol, and anthocyanins have been reported to suppress adipogenesis and lipogenesis through modulation of PPAR gamma and C/EBP alpha, while compounds such as curcumin, berberine, and capsaicin have demonstrated thermogenic and metabolic effects [22,23,24,25,26]. Several natural compounds also influence endocrine and appetite signaling, including GLP-1 responsiveness, and modulate gut-brain access involved in energy homeostasis [27,28,29].

Despite promising preclinical findings, translation of these bioactive compounds into effective anti-obesity interventions remains limited by poor oral bioavailability, rapid metabolism, and substantial heterogeneity in plant-derived preparations [21,28,29]. Strategies such as nanoencapsulation and phospholipid complexes may improve systemic exposure and tissue targeting; however, standardized formulations and rigorous dose-response studies remain insufficient. Also, major historical limitation in the development of nutraceuticals for obesity management has been the reliance on isolated, single-target natural compounds [21,28,29]. Although many individual phytochemicals demonstrate promising efficacy in *in vitro* screens, they frequently fail during *in vivo* translation due to the aforementioned poor systemic bioavailability, rapid metabolic clearance, and the counter-regulatory pathways activated by multifactorial diseases like obesity. Furthermore, the previous literature has often been constrained by either purely descriptive *in vitro* analyses or uncharacterized crude extracts lacking standardization, creating significant barriers to reproducibility [30]. Crucially, by shifting from a single-molecule paradigm to a multi-targeted combination, it is possible to demonstrate robust phenotypic efficacy *in vivo* at concentrations where individual components typically exhibit limited success, thereby providing a reproducible blueprint for overcoming single-compound pharmacological limitations [31,32]. Consequently, natural compounds are currently considered adjuncts rather than alternatives to evidence-based pharmacological or surgical interventions.

This gap between strong mechanistic rationale and limited translational evidence underscores the need for systematic studies investigating multi-target botanical formulations for obesity management. While individual botanical components possess documented metabolic properties, single-molecule interventions often exhibit limited efficacy in multifaceted, multifactorial disorders due to biological redundancy. Complex metabolic syndromes like obesity often require a multi-targeted therapeutic approach [30,31]. Rather than relying on a single high-dose molecule, the low-dose multi-component approach aims to leverage phytochemical multi-target interactions, potentially maximizing therapeutic efficacy across multiple tissues (enteroendocrine cells, adipocytes, and adipose tissue) while minimizing the risk of compound-specific toxicity. Accordingly, the present study aimed to identify and characterize phytochemical combinations capable of stimulating GLP-1 secretion and activating AMPK signaling *in vitro* and to determine whether coordinated modulation of these pathways confers anti-obesity effects in high-fat diet-induced obese mice. We further evaluated the effects of selected formulations on lipid accumulation, inflammatory markers, and metabolic parameters to establish the mechanistic potential of a multi-component botanical strategy for obesity management. Combination #4 was found to achieve multi-pathway target coverage by combining distinct phytochemical matrices that target separate, yet converging, physiological nodes. Specifically, while components like white kidney bean and fenugreek extracts primarily modulate carbohydrate absorption and pancreatic enzyme activity, constituents like ginseng, curcumin, bitter melon, and capsaicin act downstream on central energy-sensing networks, including GLP-1 secretion and AMPK activation.

## 2. Materials and Methods

### 2.1. Materials

#### 2.1.1. Test Compounds and Test Compositions Used in *In Vitro* and *In Vivo* Studies

Commercially available plant extracts such as African mango extract, juniper berry extract, white kidney bean extract, bitter melon extract, and fenugreek seed extract were purchased from Selleck Chemicals LLC (Houston, TX, USA) and commercially available plant extracts such as ginseng root extract, saffron extract, and ginger extract from Sigma (St. Louis, MO, USA). Chemical identity, purity, and phytochemical standardization of each extract were established based on the manufacturer’s Certificates of Analysis (CoA), technical data sheets, and/or validated analytical documentation provided by the supplier. These documents report on compound identity, marker compound quantification, and/or activity-based standardization using validated analytical methods (e.g., HPLC, infrared spectroscopy (IR), and activity assays) under the supplier’s quality assurance framework. The extracts were therefore considered standardized according to their respective characteristic phytochemical marker compounds as specified by the manufacturer, and these specifications are summarized in Table 1. Test compounds such as capsaicin, hydroxycitric acid (HCA), berberine, curcumin, and diindolylmethane (DIM) with 95–99% purity (according to the manufacturer) were obtained from Cayman Chemical (Ann Arbor, MI, USA). Formulations of test compositions are presented in Table 2.

The *in vitro* testing range for the 14 phytochemicals and standardized botanical extracts was determined using preliminary cell viability assays to confirm non-toxic parameters, alongside physiologically relevant concentrations established in published pharmacokinetic literature, and physiological bio-accessibility criteria. Furthermore, because these extracts function as luminal secretagogues directly interacting with the intestinal mucosa upon oral intake, this microgram concentration range closely reflects the expected undegraded luminal density following our oral *in vivo* doses, prior to systemic hepatic metabolism.

#### 2.1.2. Human NCI-H716 Cells Culture and Maintenance as a Model to Study GLP-1 Secretion

NCI-H716, a suspension cell line with lymphoblast morphology isolated from the cecum of a patient with colorectal adenocarcinoma and commonly used as an intestinal enteroendocrine L-cell model, was obtained from ATCC (American Type Culture Collection) (Manassas, VA, USA). This cell line was maintained in RPMI-1640 medium with 10% fetal bovine serum (FBS) at 37 °C in a humidified atmosphere with 5% CO_2_.

#### 2.1.3. Human Primary Preadipocytes Culture and Differentiation as a Model to Study Lipid Deposition

Human primary preadipocytes (ATCC, Gaithersburg, MD, USA) were cultured in Fibroblast Growth Kit-Low serum at 37 °C in a humidified atmosphere with 5% CO_2_. To differentiate preadipocytes into mature adipocytes, fully confluent preadipocytes were treated with Adipocyte Differentiation Initiation Medium with or without test compounds in different combinations as presented in Table 2 (defined as Day 0). After 48 h of incubation, the Adipocyte Differentiation Initiation Medium was replaced with Adipocyte Differentiation Maintenance Medium in the presence or absence of test compounds in different combinations (Day 2) for another six days (Day 8).

### 2.2. Methods

#### 2.2.1. GLP-1 *In Vitro* Secretion Assay

Human NCI-H716 cells were treated for 16 h with individual test compounds or their corresponding combinations. Following treatment, conditioned medium was collected and analyzed for GLP-1 secretion using a human GLP-1 ELISA kit (MyBioSource, San Diego, CA, USA) according to the manufacturer’s instructions. Absorbance was measured at 450 nm using a microplate reader (Tecan, Mannedorf, Switzerland). Results are expressed as a percentage of vehicle-treated control (mean ± SD, *n* = 3).

#### 2.2.2. Oil-Red O Staining

Intracellular lipid accumulation in differentiated adipocytes was assessed using an Oil-Red O staining kit (Lifeline Cell Technology, San Diego, CA, USA) according to the manufacturer’s protocol. On Day 8 of differentiation, adipocytes were washed with phosphate-buffered saline (PBS) and fixed with 4% paraformaldehyde for 1 h at room temperature. Cells were then stained with Oil-Red O solution for 4 h. After staining, dye was extracted using 100% 1,2-propanediol, and absorbance was measured at 520 nm using a microplate reader (Tecan, Mannedorf, Switzerland). Results are expressed as a percentage of vehicle-treated control (mean ± SD, *n* = 4).

#### 2.2.3. MCP-1 *In Vitro* Secretion Assay

Differentiated human adipocytes cultured in 6-well plates were treated with the indicated test combinations for 16 h. Conditioned medium was collected, centrifuged at 12,000 rpm for 5 min to remove cellular debris, and analyzed using a human MCP-1 ELISA kit (Thermo Fisher Scientific, Waltham, MA, USA) according to the manufacturer’s instructions. Absorbance was measured at 450 nm using a microplate reader (Tecan, Mannedorf, Switzerland). Results are expressed as a percentage of vehicle-treated control (mean ± SD, *n* = 3).

#### 2.2.4. pAMPK *In Vitro* Assay

Differentiated human adipocytes were treated with the indicated test combinations for 16 h in 6-well plates. Cells were lysed using RIPA buffer supplemented with protease and phosphatase inhibitor cocktails. Phosphorylated AMPK (pAMPK) levels were quantified using a pAMPK ELISA kit (Thermo Fisher Scientific, Waltham, MA, USA) according to the manufacturer’s instructions. Absorbance was measured at 450 nm using a microplate reader (Tecan, Mannedorf, Switzerland). Results are expressed as a percentage of vehicle-treated control (mean ± SD, *n* = 3).

#### 2.2.5. Viability Assay

Cell viability was assessed using the 3-(4,5-dimethylthiazol-2-yl)-2,5-diphenyl tetrazolium bromide (MTT) assay. Human NCI-H716 cells were seeded into 96-well plates at a density of 4 × 10^4^ cells/well and incubated overnight prior to treatment. Cells were then exposed to individual test compounds or their combinations at concentrations of 10 or 20 µg/mL for 24 h. Following treatment, culture medium was replaced with fresh medium containing 5 mg/mL MTT reagent, and cells were incubated for an additional 3 h at 37 °C. After removal of the medium, formazan crystals were dissolved in 100 µL methanol, and absorbance was measured at 570 nm using a microplate spectrophotometer (Molecular Devices, San Jose, CA, USA). Results are expressed as a percentage of vehicle-treated control (mean ± SD, *n* = 8).

#### 2.2.6. *In Vivo* Study

Animal experimental procedures were approved by the Institutional Animal Care and Use Committee (IACUC) of Dr. Rath Research Institute (San Jose, CA, USA) (protocol No.: 08/B012025 approved on 1 October 2025). This study is reported in accordance with ARRIVE guidelines. Male C57BL/6J mice at four weeks old were obtained from Charles River Laboratories (Palo Alto, CA, USA). The Gulo (−/−);hLp-a+ strain (Lp(a) mice) was developed by Dr. Rath Research Institute and is currently in its possession. Both strains of mice were maintained in controlled conditions (temperature of 22.0 ± 2.0 °C, humidity of 50.0 ± 2.0%, and a 12 h light/dark cycle). During acclimatization for one week, mice were given free access to water and food (C57BL/6J strain: #5001 standard rodent diet, Gulo (−/−);hLp-a+ strain: #5001 standard rodent diet with 1% vitamin C, both from TestDiet, Richmond, IN). After acclimatization, mice were fed either a normal-fat diet (ND, n = 8) as a control group or a high-fat diet (HFD, n = 16) (#58Y1 rodent diet with 60% kcal from fat, TestDiet, Richmond, IN, USA) as an HFD-induced obesity group for five weeks. After obesity induction, obese mice were further divided into the following two experimental groups (*n* = 8/group) and matched by body weight: HFD group and HFD + A (high-fat diet + 0.5% additives, 500 mg/kg). All additives/components of the botanical formulation (i.e., combination 4, Table 3) were commercially obtained and utilized without further purification. The formulation consisted of four standardized botanical extracts (ginseng root extract, white kidney bean extract, bitter melon extract, and fenugreek seed extract) and two highly purified phytochemical compounds (curcumin and capsaicin, both 95% and 98% purity). The botanical nomenclature, manufacturer identifiers, primary marker compounds, and specific standardization or purity thresholds for each component are comprehensively detailed in Table 1. Phytochemical specifications and batch authentication were established based on the validated analytical traces, technical data sheets, and CoA provided by the supplier. The animal groups were monitored for six weeks. Body weight, food, and water intake were measured twice a week after group separation. At the end of the experiment, mice were euthanized. Blood was collected, and WATs were rapidly removed, rinsed with physiological saline solution, weighed, and stored at −80 °C.

For the *in vivo* study, murine dosage was translated from human equivalents using a body surface area (BSA) normalization formula that accounts for the baseline metabolic rate of mice, outlined by Reagan-Shaw et al. [33]. The human equivalent dose (HED) was calculated as: HED = Animal Dose (mg/kg) × (Mouse Km/Human Km); Total dose = 500 mg/kg × (3/37) = 40.54 mg/kg. For a standard 70 kg human adult, this translates to a daily oral intake of approximately 2.8 g. Establishing a clear translational bridge between *in vitro* cell models and *in vivo* metabolic outcomes requires robust dose justification. In this study, the non-toxic screening envelope defined by our 24 h MTT assay (5–20 μg/mL) corresponds with the localized luminal environments encountered by enteroendocrine L-cells during our 30–100 mg/kg oral dosing protocols in C57BL/6 and Gulo (−/−);hLp-a+ mice. Because gut-restricted enteroendocrine cells directly sample chyme, they are exposed to high localized concentrations of oral extracts before any systemic dilution occurs. The preservation of cellular health over 24 h *in vitro*, combined with robust active GLP-1 secretagogue efficacy, supports the physiological and translational validity of using Composition #4 as a multi-component oral therapeutic for metabolic disorders.

#### 2.2.7. Biochemical Analysis *In Vivo*

The blood collected before autopsy was stabilized in a Microtainer^®^ blood collection tube, SST (Becton Dickinson, Fort Lauderdale, FL, USA). Serum was prepared after centrifugation at 5000 rpm for 5 min. at 4 °C and then stored at −80 °C until use. Lysates from adipocyte tissue were prepared with 5.0 mg of tissue treated with T-PER Tissue Protein Extraction Reagent supplemented with protease inhibitors cocktail and phosphatase inhibitors cocktail (ThermoFisher, Waltham, MA, USA). The contents of HbA1c were analyzed using the HbA1c ELISA kit (CristalChem, Elk Grove Village, IL, USA). Briefly, 112 µL of protease buffer was added to 5.0 µL of blood, followed by adding 48 µL of buffer 1B. Next, 25 µL of such a prepared sample was added to a 96-well plate, incubated for 5 min at 37 °C and OD measurement at 700 nm. Next, 70 µL enzyme solution was added, and the sample was again incubated for 3 min at 37 °C. Then, the final OD measurement at 700 nm was performed, using a microplate reader (Tecan, Mannedorf, Switzerland). Results are expressed as a percentage of experimental control (i.e., ND study group, mean ± SD, *n* = 8). The contents of obesity-relevant biochemical parameters were also analyzed and performed by a third party, Inotiv (Boulder, CO, USA). The contents of the MCP-1, insulin, leptin, GLP-1, and GLP-1 receptor (GLP-1R) in serum and/or subcutaneous adipocyte tissue were analyzed using an enzyme-linked immunosorbent assay (ELISA) kit performed as described below:

(A) Insulin assay: Insulin levels in mouse serum (5× dilution) were evaluated using an insulin ELISA kit (MyBioSource, San Jose, CA, USA). Briefly, 50 µL of diluted serum was added to each 96-well pre-coated plate, followed by adding 100 µL of HRP-conjugate reagent and 1 h incubation at 37 °C. Next plate was 3× washed with 1× washing buffer, and 50 µL of chromogen solution A and 50 µL of chromogen solution B were added, and the plate was again incubated for 15 min at 37 °C. Reaction was stopped by adding 50 µL of stop solution, and OD measurement at 450 nm was performed immediately, using a microplate reader (Tecan, Mannedorf, Switzerland). Results are expressed as a percentage of the experimental control group ND (mean ± SD, *n* = 8).

(B) Leptin assay: Leptin levels in mouse serum (5× dilution) were evaluated using a leptin ELISA kit (Abcam, Waltham, MA, USA). Briefly, 50 µL of diluted serum was added to each 96-well pre-coated plate, followed by adding 50 µL of antibody cocktail and 1 h incubation at room temperature on a plate shaker set to 400 rpm. Next plate was 3× washed with 1× washing buffer. Then, 100 µL of TMB development solution was added, and the plate was again incubated for 10 min at room temperature on a plate shaker set to 400 rpm. Reaction was stopped by adding 100 µL of stop solution, and OD measurement at 450 nm was performed immediately, using a microplate reader (Tecan, Mannedorf, Switzerland). Results are expressed as a percentage of the experimental control group ND (mean ± SD, *n* = 8).

(C) MCP-1 assay: MCP-1 levels in mouse serum (5× dilution) were evaluated using an MCP-1 ELISA kit (Abcam, Waltham, MA, USA). Briefly, 50 µL of diluted serum was added to each 96-well pre-coated plate, followed by adding 50 µL of antibody cocktail and 1 h incubation at room temperature on a plate shaker set to 400 rpm. Next plate was 3× washed with 1× washing buffer. Then, 100 µL of TMB development solution was added, and the plate was again incubated for 10 min at room temperature on a plate shaker set to 400 rpm. Reaction was stopped by adding 100 µL of stop solution, and OD measurement at 450 nm was performed immediately, using a microplate reader (Tecan, Mannedorf, Switzerland). Results are expressed as a percentage of the experimental control group ND (mean ± SD, *n* = 8).

(D) GLP-1 assay: GLP-1 levels in mouse serum (2.5× dilution) and WAT lysate were evaluated using a GLP-1 ELISA kit (MyBioSource, San Jose, CA, USA). Briefly, 50 µL of diluted serum or tissue lysate was added to each 96-well pre-coated plate, followed by adding 50 µL of HRP-conjugate reagent and 1 h incubation at 37 °C. Next plate was 3× washed with 1× washing buffer, and 50 µL of Substrate A and 50 µL of Substrate B were added, and the plate was again incubated for 15 min at 37 °C. Reaction was stopped by adding 50 µL of stop solution, and OD measurement at 450 nm was performed immediately, using a microplate reader (Tecan, Mannedorf, Switzerland). Results are expressed as a percentage of the experimental control group ND (mean ± SD, *n* = 8).

(E) GLP-1R assay: GLP-1R levels in mouse serum (2.5× dilution) and WAT lysate were evaluated using GLP-1 receptor ELISA kit (MyBioSource, San Jose, CA, USA). Briefly, 50 µL of diluted serum or tissue lysate was added to each 96-well pre-coated plate, followed by adding 50 µL of HRP-conjugate reagent and 1 h incubation at 37 °C. Next plate was 3× washed with 1× washing buffer, and 50 µL of Substrate A and 50 µL of Substrate B were added, and the plate was again incubated for 15 min at 37 °C. The reaction was stopped by adding 50 µL of stop solution, and OD measurement at 450 nm was performed immediately using a microplate reader (Tecan, Mannedorf, Switzerland). Results are expressed as a percentage of the experimental control group ND (mean ± SD, *n* = 8).

#### 2.2.8. Histological Analysis

WATs samples were fixed in 10% neutral-buffered formalin, embedded in paraffin, sectioned at 5 µm, and mounted on glass slides. Hematoxylin and eosin (H&E) staining was performed by Inotiv (Boulder, CO, USA). Histological images were acquired using an Aperio AT2 digital slide scanner (Leica Biosystems, Buffalo Grove, IL, USA). For the quantitative assessment of adipocyte hypertrophy, H&E-stained digital images of subcutaneous adipose tissue were subjected to blinded morphometric analysis. Image files were randomized and coded by an independent investigator to eliminate observer bias. Individual adipocyte cross-sectional areas (μm^2^) were measured using ImageJ software (Version 1.54, NIH, Bethesda, MD, USA) calibrated with a pixel-to-micrometer scale bar. A minimum of 100 fully intact adipocytes was measured across at least three random, non-overlapping fields of view per animal (*n* = 8 animals per group). Adipocyte size profiles were expressed as mean cross-sectional area ± standard deviation (SD).

#### 2.2.9. Statistical Analysis

Data are presented as mean ± SD. *In vitro* experiments were performed in at least three independent biological replicates, whereas *in vivo* analyses were conducted using *n* = 8 animals per group. Statistical analyses were performed using GraphPad Prism software (GraphPad Software, Version 9.0, Boston, MA, USA). Differences in cumulative metrics isolated at the final 11-week time point were evaluated using a two-tailed Student’s *t*-test (for single-pair comparisons) or a One-Way ANOVA followed by Tukey’s post hoc analysis. To analyze chronological changes tracked twice per week over the 11-week duration (body weight gain, food intake, and water consumption), a two-way repeated-measures ANOVA was performed. When a significant main effect or interaction was detected by the ANOVA, Tukey’s post hoc multiple comparisons test was applied to calculate specific pairwise differences between treatment groups at individual time points while controlling the family-wise error rate. Differences were considered statistically significant at *p* < 0.05. Where chronological post hoc testing revealed that differences failed to reach the critical threshold, parameters were designated as statistically non-significant (*p* > 0.05) to preserve conservative interpretation boundaries.

## 3. Results

### 3.1. In Vitro Study of Complementary Activation of GLP-1 and AMPK Pathways by a Phytochemical Intervention

#### 3.1.1. Effects of Test Compounds and Test Combinations on Viability of Human NCI-H176 Cells

**Figure 1:**
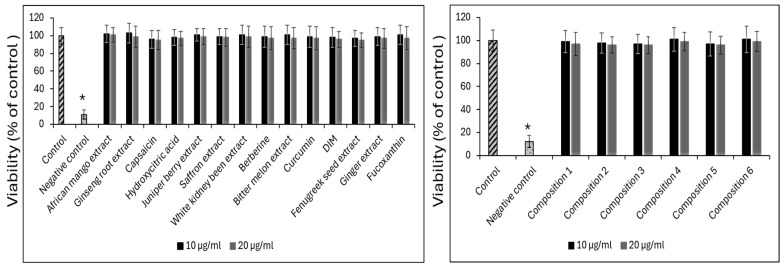
*In vitro* cytotoxic effect of individual test agents and test combinations. NCI-H716 cells were treated with 10 µg/mL and 20 µg/mL of individual agents (**left** panel) as well as their combinations (**right** panel) for 24 h, followed by the assessment of their viability using MTT method and measuring absorbance at 570 nm. For both panels, independent data subsets for each separate concentration endpoint (10 µg/mL and 20 µg/mL) were analyzed via independent One-Way ANOVAs followed by Tukey’s post hoc multiple comparisons test against the vehicle control to control the family-wise error rate across multiple parallel endpoints. Exact model parameters are as follows: for the left panel individual agent matrix (comprising vehicle control, negative control, and 14 independent test agents; k = 16), F(15, 32) = 1073.90, *p* < 0.001 at 10 µg/mL and F(15, 32) = 41.15, *p* < 0.001 at 20 µg/mL; for the right panel combination matrix (comprising vehicle control, negative control, and 6 independent test combinations; k = 8) F(7, 16) = 94.91, *p* < 0.001 at 10 µg/mL and F(7, 16) = 85.35, *p* < 0.001 at 20 µg/mL; * *p* < 0.001 compared to vehicle control; control–0.02% DMSO (vehicle control), negative control–100% dead cells. Data represent mean ± SD from *n* = 3 independent biological repetitions.

The cytotoxic effect of test compounds and test combinations on NCI-H176 cells was evaluated using the MTT assay. The results presented in Figure 1 showed that all test compounds and test combinations did not exhibit cytotoxicity at 10 µg/mL or 20 µg/mL concentrations.

#### 3.1.2. Effects of Test Compounds and Test Combinations on GLP-1 Secretion in Human NCI-H176 Cells

**Figure 2:**
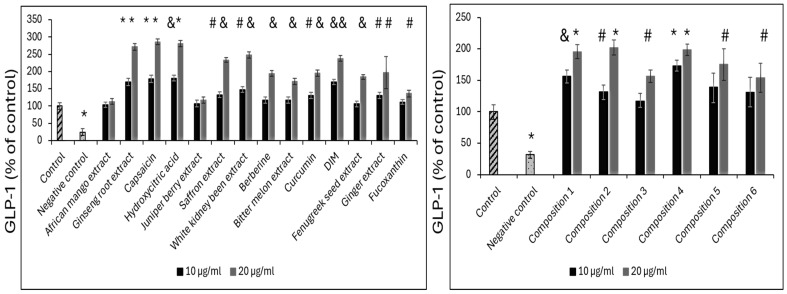
*In vitro* effect of individual test agents and test combinations on GLP-1 secretion. NCI-H716 cells were treated with individual test agents (**left** panel) or their combinations (**right** panel) at 10 µg/mL and 20 µg/mL and further subjected to a GLP-1 ELISA kit to examine levels of GLP-1 release. To track secretion changes across distinct experimental dimensions while preventing multi-endpoint Type I error rate inflation, datasets were compartmentalized into independent hypothesis families by concentration. Within each family, independent data subsets for each separate concentration endpoint (10 µg/mL and 20 µg/mL) were analyzed via separate independent One-Way ANOVAs followed by Tukey’s post hoc multiple comparisons test against the vehicle control to strictly control the family-wise error rate. Exact model parameters are as follows: for the left panel individual agent matrix (comprising vehicle control, negative control, and 14 independent test agents; k = 16), F(15, 32) = 33.04, *p* < 0.001 at 10 µg/mL and F(15, 32) = 5444.13, *p* < 0.00120 µg/mL; for the right panel combination matrix (comprising vehicle control, negative control, and 6 independent test combinations; k = 8), F(7, 16) = 36.44, *p* < 0.001 at 10 µg/mL F(7, 16) = 187.61, *p* < 0.001 at 20 µg/mL; # *p* < 0.05, & *p* < 0.01, * *p* < 0.001 compared to vehicle control; control–0.02% DMSO (vehicle control), negative control–100% dead cells. Data represent mean ± SD from *n* = 3 independent biological repetitions.

The effect of test compounds and test combinations on GLP-1 secretion was evaluated in the human enteroendocrine NCI-H176 cell line, an intestinal L-cells model, and has been used for studying gut hormonal responses to food intake and the regulation of GLP-1 secretion (Figure 2). NCI-H176 cells were treated with 10 µg/mL and 20 µg/mL concentrations of test compounds and test combinations for 16 h, and GLP-1 secretion was evaluated using a commercially available GLP-1 ELISA kit as described in the Materials and Methods. Results presented in Figure 2 (lrft panrl) show that among 14 test compounds, eight of them such as ginseng root extract, capsaicin, hydroxycitric acid, saffron extract, white kidney bean extract, berberine, curcumin, DIM, and ginger increased GLP-1 secretion ranging from 11–112% (18.9–93.3 pg/mL) at 10 µg/mL concentration with ginseng root extract, capsaicin and hydroxycitric acid having the most pronounced effects. At a 20 µg/mL concentration, twelve compounds, such as ginseng root extract, capsaicin, hydroxycitric acid, saffron extract, white kidney bean extract, berberine, bitter melon extract, curcumin, DIM, fenugreek extract, ginger, and fucoxanthin, stimulated GLP-1 secretion ranging from 24–179% (33.3–141.9 pg/mL). Moreover, the results presented in Figure 2 (right panel) reveal that among six test combinations three of them (combinations #1, #2, and #4) increased GLP-1 secretion ranging from 19–74% (24.6–68.7 pg/mL), at 10 µg/mL concentration, and all of them increased GLP-1 secretion ranging from 52–101% (62.7–81.6 pg/mL) at 20 µg/mL concentration. A key methodological consideration in this study was the reliance on the NCI-H716 cell line for GLP-1 secretion assays. As a human colorectal adenocarcinoma-derived line, NCI-H716 cells may not completely recapitulate the exact, non-transformed physiology of healthy human L-cells. Transformed cell lines can exhibit altered receptor expression densities and signaling kinetics, which represent potential model-specific artifacts. Despite these limitations, NCI-H716 cells are widely recognized as the gold standard for human-derived *in vitro* L-cell modeling because primary L-cells are extremely rare and difficult to isolate or sustain in long-term culture. Crucially, the stimulatory pathways identified in this study align with historical data from alternative enteroendocrine systems such as murine STC-1 or GLUTag lines, where similar phytochemical profiles have successfully stimulated GLP-1 release. Nevertheless, future validation using primary human intestinal organoids or ex vivo tissue models will be instrumental in confirming the translational accuracy of these findings.

#### 3.1.3. Effects of Test Combinations on Lipid Accumulation in Human Differentiated Adipocytes

**Figure 3:**
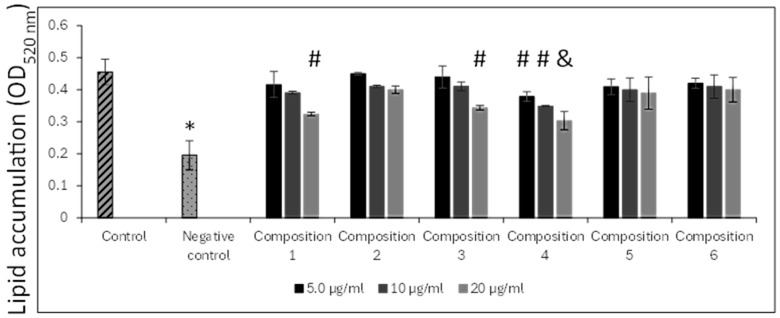
*In vitro* effect of test combinations on lipogenic responses in human differentiated adipocytes. Evaluation of lipid accumulation in human mature adipocytes treated with 5.0 µg/mL, 10 µg/mL, and 20 µg/mL of the test combination for 8 days, using an Oil-Red O staining kit and measuring absorbance at 520 nm. To evaluate lipogenic trends across distinct experimental dimensions while preventing multi-endpoint Type I error rate inflation, datasets were compartmentalized into independent hypothesis families by concentration. Within each family, independent data subsets for each separate concentration endpoint (5.0 µg/mL, 10 µg/mL, and 20 µg/mL) were analyzed via separate independent One-Way ANOVAs followed by Tukey’s post hoc multiple comparisons test against the vehicle control to strictly control the family-wise error rate. Exact model parameters are as follows: for the 5.0 µg/mL concentration family (comprising vehicle control, negative control, and 6 independent test combinations; k = 8), 5.0 µg/mL: F(7, 16) = 24.81, *p* < 0.001; 10 µg/mL: F(7, 16) = 17.74, *p* < 0.001; 20 µg/mL: F(7, 16) = 16.89, *p* < 0.001). # *p* < 0.05, & *p* < 0.01, * *p* < 0.001 compared to vehicle control; control–0.02% DMSO (vehicle control), negative control–100% dead cells. Data represent mean ± SD from *n* = 3 independent biological repetitions.

Considerable lipid accumulation that leads to adipocyte dysfunction has been widely recognized as the primary factor in the development of obesity-related metabolic syndrome. We investigated the effect of test combinations used at 5.0 µg/mL, 10 µg/mL and 20 µg/mL on lipid accumulation in human mature adipocytes developed from pre-adipocytes as described in the Materials and Methods. Lipid droplets were stained with Oil-Red O staining dye, and lipid concentrations were quantified. Figure 3 shows that only the combination #4 reduced lipid accumulation in a dose-dependent manner by 11% at 5.0 µg/mL, 26% at 10 µg/mL, and 42% at 20 µg/mL. Two other combinations, such as combination #1 and combination #3, had lesser effects by suppressing lipid accumulation by about 20–22% at 20 µg/mL.

#### 3.1.4. Effects of Test Combinations on the Level of pAMPK Protein in Human Differentiated Adipocytes

**Figure 4:**
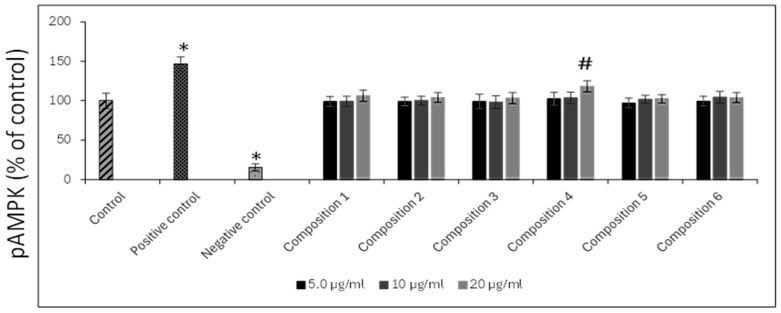
*In vitro* effect of test combinations on adipogenic responses in human differentiated adipocytes. Evaluation of pAMPK level in human mature adipocytes treated with 5.0 µg/mL, 10 µg/mL, and 20 µg/mL test combination for 16 h, using pAMPK ELISA kit and measuring absorbance at 450 nm. To evaluate kinase activation trends across distinct experimental dimensions while preventing multi-endpoint Type I error rate inflation, datasets were compartmentalized into independent hypothesis families by concentration. Within each family, independent data subsets for each separate concentration endpoint (5.0 µg/mL, 10 µg/mL, and 20 µg/mL) were analyzed via separate independent One-Way ANOVAs followed by Tukey’s post hoc multiple comparisons test against the vehicle control to strictly control the family-wise error rate. Exact model parameters are as follows: for the 5.0 µg/mL concentration family (comprising vehicle control, negative control, manufacturer-provided positive control standard, and 6 independent test combinations; k = 9), F(8, 18) = 4.71, *p* < 0.05; for the 10 µg/mL family (k = 9), F(8, 18) = 31.91, *p* < 0.001; and for the 20 µg/mL family (k = 9), F(8, 18) = 14.15, *p* < 0.001. # *p* < 0.05, * *p* < 0.001 compared to vehicle control; control–0.02% DMSO (vehicle control), negative control–100% dead cells, positive control–pAMPK protein as positive control provided by manufacturer. Data represent mean ± SD from *n* = 3 independent biological repetitions.

We evaluated the effect of test combinations on the level of pAMPK in human mature adipocytes developed from pre-adipocytes as described in the Materials and Methods. Differentiated cells were treated with test combinations at 5.0 µg/mL, 10 µg/mL, and 20 µg/mL concentrations for 16 h, and then subjected to pAMPK ELISA kit. Results presented in Figure 4 showed that among all test combinations, only the combination #4 significantly increased pAMPK level by about 21.2% (11.1 units/mL) at 20 µg/mL concentration.

#### 3.1.5. Effects of Test Combinations on Secretion Level of MCP-1 Protein in Human Differentiated Adipocytes

**Figure 5:**
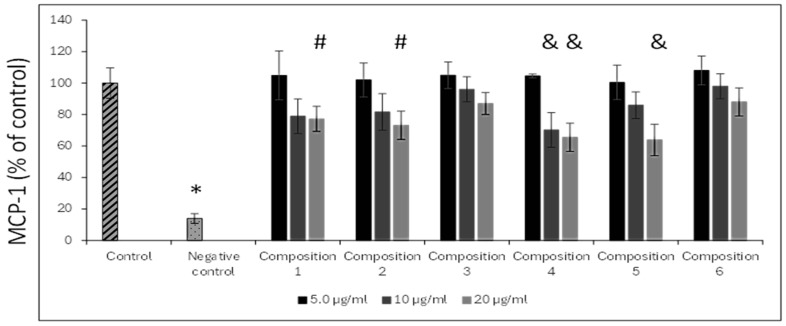
*In vitro* effect of test combinations on inflammatory responses in human differentiated adipocytes. Evaluation of MCP-1 level in human mature adipocytes treated with 5.0 µg/mL, 10 µg/mL, and 20 µg/mL test combination for 8 days, using MCP-1 ELISA kit and measuring absorbance at 450 nm. To evaluate chemokine secretion trends across distinct experimental dimensions while preventing multi-endpoint Type I error rate inflation, datasets were compartmentalized into independent hypothesis families by concentration. Within each family, independent data subsets for each separate concentration endpoint (5.0 µg/mL, 10 µg/mL, and 20 µg/mL) were analyzed via separate independent One-Way ANOVAs followed by Tukey’s post hoc multiple comparisons test against the vehicle control to strictly control the family-wise error rate. Exact model parameters are as follows: for the 5.0 µg/mL concentration family (comprising vehicle control, negative control, and 6 independent test combinations; k = 8), F(7, 16) = 136.55, *p* < 0.001; for the 10 µg/mL family (k = 8), F(7, 16) = 138.68, *p* < 0.001; and for the 20 µg/mL family (k = 8), F(7, 16) = 92.08, *p* < 0.001. # *p* < 0.001, & *p* < 0.01, * *p* < 0.001 compared to control; control–0.02% DMSO (vehicle control), negative control–100% dead cells. Data represent mean ± SD from *n* = 3 independent biological repetitions.

To investigate the effects of test combinations on the secretion of obesity-relevant cytokine MCP-1, preadipocytes were first treated with Adipocyte Differentiation Initiation Medium with or without test combinations. After two days of incubation, the Adipocyte Differentiation Initiation Medium was replaced with Adipocyte Differentiation Maintenance Medium with or without test combinations at 5.0 µg/mL, 10 µg/mL and 20 µg/mL, respectively. At the end of the experiment, conditioned media were collected and subjected to the MCP-1 ELISA kit. Figure 5 shows that all six test combinations could decrease MCP-1 secretion by different degrees in a concentration-dependent fashion. The most effective was test combination #4, which decreased the MCP-1 secretion by 29.2% when applied at a 10 µg/mL concentration and by 34.5% at 20 ug/mL. When applied at a 20 µg/mL concentration, the combinations #1, #2, and #5 decreased MCP-1 secretion by 22.8%, 26.9%, and 36.1%, respectively, while combinations #3 and #6 were the least effective.

### 3.2. In Vivo Study of Multi-Component Nutritional Formulation for Synchronized Anti-Obesity Efficacy

#### 3.2.1. Effects of Test Combination #4 on Body Weight and Food and Water Intake in Mice with High-Fat Diet-Induced Obesity

**Figure 6:**
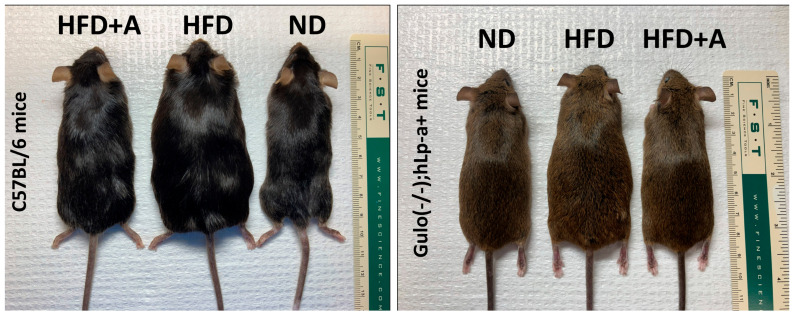
Effect of test combination #4 on obesity status in mice. Representative images of C57BL/6 mice (**right** images) and Gulo (−/−);hLp-a+ mice (**left** images) fed normal diet (*n* = 8/group), high-fat diet (*n* = 8/group) or high-fat diet + additives (*n* = 8/group) at the end of experiment (11 weeks). ND—normal diet, HFD—high-fat diet, HFD + A—high-fat diet + additives.

Since combination #4 displayed the most optimal balance of high metabolic efficacy and low cytotoxicity, it was prioritized for subsequent *in vivo* validation (Table S1). The effect of composition #4 on weight as well as food and water intake in HFD-induced obesity in two strains of mice: C57BL/6 and Gulo (−/−);hLp-a+ was investigated. As shown in Figure 6 and Table 4, at the end of the experiment (11 weeks), the body weight gain of the HFD groups significantly increased (102.9% in the C57BL/6 strain, 78.4% in the Gulo (−/−);hLp-a+ strain) when compared with the ND groups, respectively. In contrast, the administration of composition #4 for 6 weeks at 500 mg/kg resulted in a significant decrease in body weight (57.8% in C57BL/6 strain, 41.8% in Gulo (−/−);hLp-a+ strain) compared to mice with HFD-induced obesity. In comparison to ND groups, mice from HFD + A groups gained about 26.9% weight for the C57BL/6 strain and 10.4% for the Gulo (−/−);hLp-a+ strain. In comparison to mice from the HFD group, mice from the HFD + A groups showed a decrease in body weight by about 28.0% in the C57BL/6 strain and about 27.0% in the Gulo (−/−);hLp-a+ strain. No significant change in water intake was noticed, and intake of food decreased by 17% and 22% in C57BL/6 and Gulo (−/−);hLp-a+ mice to the similar levels observed in ND groups, respectively (Table 5). In addition, we have performed a comprehensive energy intake analysis by calculating the Feed Efficiency Ratio (FER) over the 77-day experimental period across our groups. The FER evaluates the efficiency of the animals in converting energy intake into body mass, using the following established formula: FER = Body Weight Gain (mg)/Total Energy Intake (kcal). Our calculations reveal a statistically distinct profile between our high-fat diet experimental cohorts. The HFD C57BL/6 group exhibited an FER of 14.50 mg/kcal (gaining 21.1 g of body weight over a total energy intake of 1455.3 kcal), while the treated HFD + A C57BL/6 group exhibited a significantly lower FER of 9.87 mg/kcal (gaining 11.9 g of body weight over a total energy intake of 1205.8 kcal). This substantial 31.9% reduction in Feed Efficiency Ratio between the two groups on the identical diet (5.4 kcal/g) indicates that the treated mice gained significantly less body mass per kilocalorie consumed compared to the HFD controls, providing supportive, indirect evidence of altered energy utilization. The HFD Gulo (−/−);hLp-a+ group exhibited an elevated FER of 13.38 mg/kcal (gaining 17.8 g of body weight over a total energy intake of 1330.6 kcal), reflecting pathological fat accumulation per calorie consumed, while the treated HFD + A Gulo (−/−);hLp-a+ group exhibited a lower FER of 9.04 mg/kcal (gaining 9.4 g of body weight over a total energy intake of 1039.5 kcal). Crucially, the FER of the HFD + A group was not only reduced by 32.4% compared to the HFD control, but it also slightly dropped below the baseline ratio of the lean normal diet (ND) group (9.68 mg/kcal).

#### 3.2.2. Effects of Test Combination #4 on WATs in Mice with High-Fat Diet-Induced Obesity

**Figure 7:**
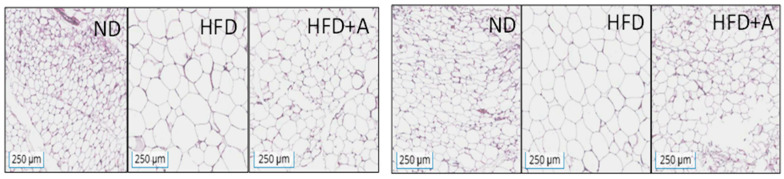
Effect of test combination #4 on WATs status in mice. Representative images of subcutaneous adipocyte tissue collected from C57BL/6 mice (**right** images) and Gulo (−/−);hLp-a+ mice (**left** images) fed normal diet (*n* = 8/group), high-fat diet (*n* = 8/group), or high-fat diet + additives (*n* = 8/group). Tissues were stained with H&E and images were taken at 200× magnification using an Aperio AT2 digital imaging system; scale bar = 250 μm; ND—normal diet, HFD—high-fat diet, HFD + A—high-fat diet + additives.

H&E staining results of WATs showed that the size of adipocytes in both strains of mice was visibly larger in HFD groups and noticeably decreased in HFD + A groups, as presented in Figure 7, Supplementary Materials and Table 6. Histological assessment of adipose tissue that initially suggested prominent visual differences in lipid droplet sizes was subsequently validated through blinded digital morphometry. In the C57BL/6 mouse cohort, the untreated HFD group developed severe adipocyte hypertrophy, showing an elevated cross-sectional area of 19,630.5 ± 2573.8 μm^2^ compared to control mice on ND (1990.2 ± 187.2 μm^2^, *p* < 0.001). Notably, intervention with composition #4 significantly reduced this hypertrophic expansion, containing the mean area to 9890.5 ± 502.8 μm^2^ (*p* < 0.001) compared to the HFD group. A mirroring and highly robust protective profile was observed within the Gulo (−/−);hLp-a+ mouse background as well. The untreated HFD Gulo (−/−);hLp-a+ mice developed pronounced cellular swelling, with the mean cross-sectional adipocyte area reaching 23,006.8 ± 2164.4 μm^2^ compared to their respective lean control mice (3875.2 ± 261.9 μm^2^, *p* < 0.001). Treatment with composition 4 remarkably suppressed this expansion in Gulo (−/−);hLp-a+ mice, limiting the mean adipocyte area to 7922.4 ± 598.9 μm^2^ (*p* < 0.001) compared to HFD mice. These quantitative outcomes demonstrate that the multi-component strategy effectively counteracts pathological adipose tissue expansion across distinct genetic models, shifting the tissue architecture by decreasing the proportion of large, hypertrophic adipocytes and increasing the prevalence of smaller, metabolically active adipocytes.

Subcutaneous adipose tissue, visceral adipose tissue, and mesenteric adipose tissue weights were also evaluated to determine the effect of test combination #4 on organ weights. As shown in Table 6 the weight of WATs significantly increased in the HFD groups compared to ND groups (subcutaneous adipose tissue weight increased by about 183.3% in C57BL/6 strain and 93.3% in Gulo (−/−);hLp-a+ strain, visceral adipose tissue weight increased 185.7% in C57BL/6 strain and 400% in Gulo (−/−);hLp-a+ strain, mesenteric adipose tissue weight increased 300% in C57BL/6 strain and 400% in Gulo (−/−);hLp-a+ strain). In contrast, the weight of WATs significantly decreased in the HFD + A groups compared to HFD groups (subcutaneous adipose tissue weight decreased by about 100.0% in C57BL/6 strain and 61.1% in Gulo (−/−);hLp-a+ strain, visceral adipose tissue weight decreased by about 66.7% in C57BL/6 strain and 120.0% in Gulo (−/−);hLp-a+ strain, mesenteric adipose tissue weight decreased by about 400% in C57BL/6 strain and 166.7% in Gulo (−/−);hLp-a+ strain). In comparison to ND groups, subcutaneous and mesenteric adipose tissues of HFD + A groups, respectively, in general showed similar values except for visceral fat weight, which was still statistically elevated by about 71.4% in the C57BL/6 strain and 66.7% in the Gulo (−/−);hLp-a+ strain.

**Figure 8:**
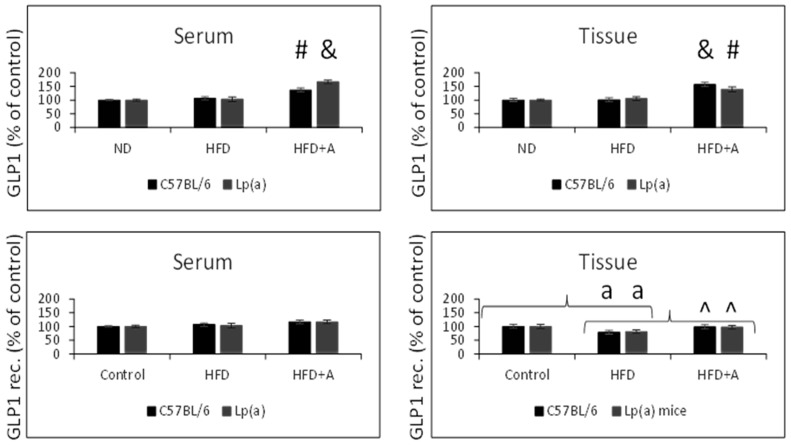
Effect of test combination #4 on GLP-1 and GLP-1R status in mice. (**Upper** panel) Levels of GLP-1 in sera and adipocyte tissue of C57BL/6 mice and Gulo (−/−);hLp-a+ mice fed normal diet (*n* = 8/group), high-fat diet (*n* = 8/group) or high-fat diet + additives (*n* = 8/group). (**Lower** panel) Levels of GLP-1 receptor in sera and adipocyte tissue of C57BL/6 mice and Gulo (−/−);hLp-a+ mice fed normal diet (*n* = 8/group), high-fat diet (*n* = 8/group) or high-fat diet + additives (*n* = 8/group). ND—normal diet, HFD—high-fat diet, HFD + A—high-fat diet + additives. Independent cross-sectional datasets for each respective mouse strain, protein target, and tissue compartment were analyzed via separate independent One-Way ANOVAs, consistently yielding highly significant main treatment effects across a uniform design variance profile of F(2, 21) for all evaluated matrices (*p* < 0.05). Specific pairwise changes between columns were isolated using Tukey’s post hoc multiple comparisons test. Significance thresholds are marked on the charts as follows: # *p* < 0.05, & *p* < 0.01 between ND and HFD + A; ^a^ *p* < 0.05 between ND and HFD; ^ *p* < 0.05 between HFD and HFD + A.

Our evaluation also included the status of GLP-1 and its receptor, GLP-1R, as well. The results shown in Figure 8 indicate that in sera of both strains of mice the levels of GLP-1 and GLP-1R in HFD groups were not changed, while in HFD + A groups GLP-1 levels were elevated about 24.9% (135.5 pg/mL) in C57BL/6 strain and about 48.7% (143.0 pg/mL) in Gulo (−/−);hLp-a+ strain, and GLP-1R levels were unchanged. Moreover, in subcutaneous WATs of the HFD groups, GLP-1 levels were unchanged, while GLP-1R were lower by about 18.1% in the C57BL/6 strain and 23.6% in the Gulo (−/−);hLp-a+ strain. Interestingly, GLP-1 levels in HFD + A groups were elevated by about 50.2% (22.5 pg/mL) in the C57BL/6 strain and 37.9% (20.7 pg/mL) in the Gulo (−/−);hLp-a+ strain, while levels of GLP-1R were restored to the levels observed in control ND groups.

#### 3.2.3. Effects of Test Combination#4 on Serum Biochemical Parameters in Mice with High-Fat Diet-Induced Obesity

Serum biochemical parameters, including glucose, total cholesterol, triglycerides, low-density lipoprotein cholesterol (LDL), high-density lipoprotein cholesterol (HDL), alanine transaminase (ALT), aspartate transaminase (AST), gamma-glutamyl transferase (GGT), blood urea nitrogen (BUN), and lipase, were investigated. As shown in Table 7, compared to ND control groups, glucose levels were elevated in HFD groups only, but the hemolysis index was at normal levels. Liver and kidney biomarkers of cytotoxicity stayed unchanged; however, lipid markers were affected. As such, lipemia indices were elevated in HFD groups only, as well as total cholesterol, LDL, triglycerides and lipase. Serum HDL levels also increased in HFD groups but stayed augmented in HFD + A.

**Figure 9:**
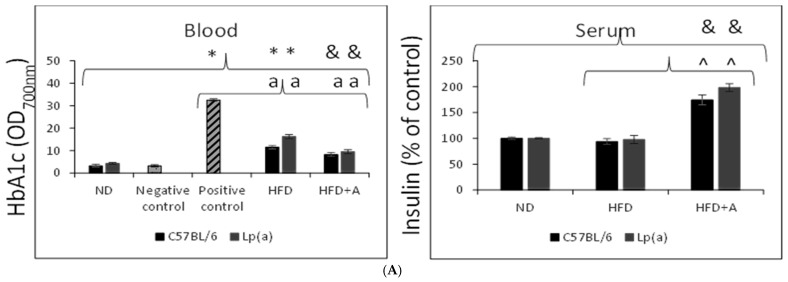

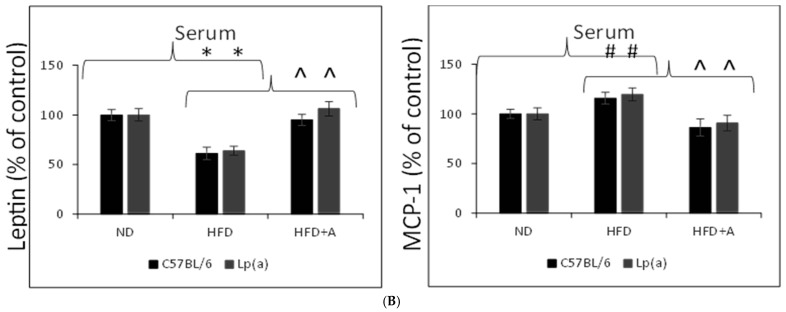
(**A**). Effect of test combination #4 on obesity-relevant parameters in mice. (**Left** panel) Levels of glycated hemoglobin HbA1c in blood of C57BL/6 mice and Gulo (−/−);hLp-a+ mice fed normal diet (*n* = 8/group), high-fat diet (*n* = 8/group) or high-fat diet + additives (*n* = 8/group). (**Right** panel) Levels of insulin in sera of C57BL/6 mice and Gulo (−/−);hLp-a+ mice fed normal diet (*n* = 8/group), high-fat diet (*n* = 8/group) or high-fat diet + additives (*n* = 8/group). ND—normal diet, HFD—high-fat diet, HFD + A—high-fat diet + additives. Independent cross-sectional datasets for each respective mouse strain and blood/serum compartment were analyzed via a One-Way ANOVA followed by Tukey’s post hoc multiple comparisons test to evaluate all pairwise interactions. Main treatment effects were highly significant, yielding design variance profiles of F(3, 28) for the 4-group HbA1c matrix (*p* < 0.05) and F(2, 21) for the 3-group insulin matrix (*p* < 0.05). Significance thresholds are marked on the charts as follows: & *p* < 0.01, * *p* < 0.001 between ND and HFD or HFD + A; ^ *p* < 0.05, between HFD and HFD + A; ^a^ *p* < 0.001 between positive control (HbA1c provided by the manufacturer) and HFD or HFD + A. (**B**). Effect of test combination #4 on obesity-relevant parameters in mice. (**Left** panel) Levels of leptin in sera of C57BL/6 mice and Gulo (−/−);hLp-a+ mice fed normal diet (*n* = 8/group), high-fat diet (*n* = 8/group) or high-fat diet + additives (*n* = 8/group). (**Right** panel) Levels of MCP-1 in sera of C57BL/6 mice and Gulo (−/−);hLp-a+ mice fed normal diet (*n* = 8/group), high-fat diet (*n* = 8/group) or high-fat diet + additives (*n* = 8/group). ND—normal diet, HFD—high-fat diet, HFD + A—high-fat diet + additives. Independent cross-sectional datasets for each respective mouse strain and serum compartment were analyzed via separate independent One-Way ANOVAs, consistently yielding highly significant main treatment effects across a uniform design variance profile of F(2, 21) for all evaluated matrices (*p* < 0.05). Specific pairwise changes between columns were isolated using Tukey’s post hoc multiple comparisons test. Significance thresholds are marked on the charts as follows: # *p* < 0.05, * *p* < 0.001 between ND and HFD; ^ *p* < 0.05 between HFD and HFD + A.

Figure 9A (left panel) shows that HbA1c levels in the blood of both strains of mice were significantly elevated in HFD groups by about 3.2-fold (8.96% HbA1c) in the C57BL/6 strain and 3.6-fold (9.72% HbA1c) in Gulo (−/−);hLp-a+ strain compared to their respective ND groups. In HFD + A groups, these markers were elevated by about 2.5-fold (7.0% HbA1c) in the C57BL/6 strain and 2.1-fold (5.67% HbA1c) in Gulo (−/−);hLp-a+ strain compared to their respective ND groups. However, HbA1c levels in HFD + A groups were moderately but significantly lower when compared to their respective HFD groups. Serum insulin level (Figure 9A, right panel) was not elevated in HFD groups compared to their respective ND groups, while in HFD + A groups, insulin level increased by about 67% (4.5 ng/mL) in the C57BL/6 strain and 84% (4.6 ng/mL) in Gulo (−/−);hLp-a+ strain compared to their respective ND groups.

Moreover, the administration of test combination #4 resulted in reduced levels of leptin in HFD groups (about 41% (2.5 ng/mL) in C57BL/6 strain and 38% (2.3 ng/mL) in Gulo (−/−);hLp-a+ strain) compared to their respective ND control groups while in HFD + A groups leptin levels were restored to their respective values in ND control groups (Figure 9B, left panel). MCP-1 levels were elevated by about 17.2% (1525 ng/mL) in the C57BL/6 strain and 21.2% (1575 ng/mL) in the Gulo (−/−);hLp-a+ in HFD groups, while in HFD + A groups, they decreased to the levels observed in control ND groups (Figure 9B, right panel).

## 4. Discussion

Obesity is a highly prevalent, chronic disease driven by interactions between biological susceptibility and obesogenic environments, with major adverse consequences for cardiometabolic health. Although excess adiposity ultimately reflects a sustained positive energy balance, its pathophysiology is largely determined by neuroendocrine, genetic, and metabolic mechanisms that promote hyperphagia, reduce energy expenditure, and defend an elevated fat mass once established [34,35].

In this work, we systematically evaluated individual compounds and their combinations for effects on GLP-1 secretion, lipid accumulation, and pAMPK level. We first selected agents with documented or anticipated anti-obesity or GLP-1–modulating properties and tested them *in vitro* in a consistent screening platform. This strategy allowed us to identify agents such as ginseng root extract, curcumin, capsaicin, white kidney bean extract, fenugreek seed extract, African mango extract, bitter melon extract, fucoxanthin, DIM, and hydroxycitric acid that were next tested in different combinations to select the one with the most effective anti-obesity metabolic effects. The series of evaluations resulted in the selection of combination #4. Specifically, while the other five candidate combinations displayed either insufficient metabolic efficacy *in vitro* (e.g., lower GLP-1 induction or minor lipid accumulation reduction) or unfavorable cytotoxicity trends at active doses, combination #4 showed the most robust, balanced, and coherent profile and was therefore advanced to *in vivo* testing. Across cellular and two animal models, combination #4 was associated with multi-factorial benefits on obesity-related phenotypes, supporting its potential as an adjunct intervention for diet-induced metabolic dysfunction and backing further investigation in clinical studies. To explore a multi-target strategy, the formulated intervention incorporates distinct bioactive compounds intended to target independent, non-overlapping pathophysiological pathways. White kidney bean extract exerts targeted enzymatic inhibition on α-amylase to modulate carbohydrate absorption, while Panax ginseng serves as a systemic metabolic modulator. Simultaneously, curcumin mitigates chronic low-grade inflammation via distinct cellular signaling cascades, and capsaicin accelerates energy expenditure through TRPV1-mediated thermogenesis. While this multi-target profile was designed to conceptually address separate, parallel facets of the underlying pathology, the precise individual contributions of each constituent remain unverified. Because formal component-deconvolution experiments, factorial designs, or synergistic interaction analyses were not performed, we cannot conclude that the therapeutic efficacy relies on a coordinated network mechanism, and the possibility remains that a single component or a small subset of ingredients drives the majority of the observed efficacy. Consequently, this combination represents a multi-ingredient formulation framework rather than an experimentally demonstrated synergistic network effect.

In our *in vitro* study, combination #4 increased GLP-1 secretion and modulated key nodes of energy and inflammatory signaling, as indicated by reduced lipid accumulation, decreased MCP-1 secretion, and increased pAMPK level. The *in vivo* experiments in HFD-induced obese C57BL/6 mice and Gulo (−/−);hLp-a+ mice corroborated these mechanistic findings. Combination #4 attenuated HFD-induced weight gain without affecting water intake and slightly lowering food intake, consistent with acknowledged GLP-1 efficacy, which might suggest effects on appetite regulation and/or nutrient utilization rather than nonspecific toxicity. Moreover, the observation of decreased leptin level in HFD mice that was restored in HFD + A mice to control levels (i.e., ND study groups) is also interesting. Normalization of fasting glucose, lipids and lipemia index, liver enzymes, and MCP-1, as well as a decrease in glycated hemoglobin HbA1c levels in HFD + A, indicated rather broad improvement in systemic metabolic control and low-grade inflammation. However, the noticably unchanged insulin level in HFD groups and the increased level of insulin in HFD + A groups are striking. Moreover, lipase levels decreased in HFD mice but restored to control levels without observed changes in GGT (i.e., marker of hepatoxicity) and BUN (i.e., marker of nephrotoxicity), further supporting this conclusion and reiterating the lack of toxicity *in vivo*. In HFD obese mice, reduced leptin levels together with unchanged insulin levels may indicate impaired adipocyte endocrine function and disrupted metabolic regulation associated with chronic high-fat feeding. MCP-1 levels were elevated in HFD mice as well, reflecting increased obesity-associated inflammation. Treatment with composition #4 restored leptin levels to those of control mice and reduced MCP-1 levels, suggesting improvements in adipose tissue function and attenuation of inflammatory responses. Although insulin levels increased in the HFD + A groups, the concomitant reduction in glycated hemoglobin (HbA1c) could indicate improved long-term glycemic control, suggesting that composition #4 may enhance insulin secretion and/or insulin sensitivity, thereby improving glucose utilization despite elevated circulating insulin.

A notable feature of our chronic 77-day high-fat diet model was the manifestation of phenotypic trends that diverge from human metabolic syndrome archetypes but tightly align with established C57BL/6 rodent biology. For instance, the unchanged insulin levels observed in our obese HFD controls may hypothetically reflect the transition to pancreatic beta-cell exhaustion, or ‘islet burnout, following prolonged lipotoxicity as documented in similar chronic C57BL/6 models. Conversely, the elevated insulin levels in the treated group, despite a significant reduction in adiposity, could theoretically align with an incretin-like cascade, as GLP-1 pathway activation characteristically promotes glucose-dependent insulin secretion. However, because direct pancreatic tissue phenotyping and dynamic endocrine assessments were outside the scope of this study, these literature-guided interpretations remain post hoc hypotheses rather than direct experimental conclusions. Furthermore, the elevation of HDL in HFD animals is a documented feature of rodent lipoprotein metabolism, as C57BL/6 mice predominantly utilize HDL to transport dietary lipids. Finally, the suppression of circulating lipase potentially mirrors subclinical pancreatic acinar dysfunction induced by chronic lipotoxic stress, which coincided with a recovery in our formulation group.

Histology and depot-specific tissue weights showed reduced adipocyte hypertrophy and decreased subcutaneous, visceral, and mesenteric fat mass toward lean levels, indicating a genuine reduction in adiposity. Furthermore, elevated levels of GLP-1 in sera and subcutaneous adipose tissues, as well as restored levels of GLP-1 receptor in subcutaneous white adipose tissue of HFD + A mice that were slightly decreased in HFD mice, together reveal a notable association with local remodeling of adipose tissue signaling, which may parallel an interruption in the feed-forward cycle linking adipose inflammation, impaired incretin action, and metabolic deterioration. To strengthen this point, across both genetic backgrounds on an identical high-fat diet (HFD; 5.4 kcal/g), the treatment consistently induced a significant ~32% reduction in the FER. This uniform drop demonstrates that treated mice stored significantly less energy as body mass per kilocalorie consumed compared to their respective HFD controls. While FER is a composite metric that cannot independently differentiate among altered intestinal absorption, nutrient partitioning, physical activity, or metabolic thermogenesis, this substantial reduction serves as supportive, indirect evidence of a treatment-induced shift in systemic energy balance. Furthermore, these *in vivo* data reinforce our *in vitro* findings, indicating that the observed alterations in the GLP-1/AMPK axes mirror a metabolic reprogramming pattern toward enhancing energy-dissipating pathways, such as thermogenesis or lipid oxidation, independent of calorie restriction alone. Notably, our *in vitro* screening demonstrated a statistically significant but modest 21% increase in pAMPK levels via ELISA. We emphasize that statistical significance does not automatically translate to biologically meaningful pathway activation, and because we did not evaluate direct downstream functional targets, such as Acetyl-CoA Carboxylase (ACC) phosphorylation, the physiological relevance of this modest *in vitro* shift remains unverified. However, important limitations of the present work include the absence of direct *in vivo* validation of the AMPK axis via Western blot, as well as the lack of direct physiological assessments of energy expenditure, such as indirect calorimetry, pair-feeding protocols, or activity monitoring. Additionally, a primary limitation of our *in vivo* metabolic phenotyping is the absence of dynamic tolerance testing, including Glucose Tolerance Tests (GTTs) and Insulin Tolerance Tests (ITTs), as well as HOMA calculations and direct pancreatic β-cell histological assessments. Without these functional evaluations, our interpretations regarding pancreatic burnout, incretin-like activity, and systemic insulin sensitivity remain literature-based hypotheses. Consequently, any exact conclusions regarding an AMPK-mediated mechanism behind the formulation’s *in vivo* effects remain tentative, and the underlying mechanisms governing the glucose-insulin dynamics are yet to be established. Future studies incorporating direct metabolic cage monitoring, dynamic GTT/ITT assays, pancreatic immunohistochemistry, and tissue-specific Western blots for both pAMPK and *p*-ACC are required to definitively map tissue-specific AMPK phosphorylation dynamics and isolate the precise components of energy expenditure modulated by this formulation. Furthermore, comprehensive component-deconvolution and factorial interaction studies are necessary to systematically isolate the individual or combinatorial efficacy of each bioactive constituent and rule out the presence of a single dominant agent. Importantly, while we observed concurrent increases in circulating GLP-1 levels and adipose GLP-1R expression alongside metabolic improvements, these findings remain strictly correlative. Because genetic loss-of-function models or receptor antagonists were not employed *in vivo*, the functional necessity of the GLP-1 signaling pathway and the actual activation state of the receptors remain to be established. Overall, the findings are consistent with a possible association involving enteroendocrine pathways, resulting in improved weight control and metabolic health under HFD conditions.

The observation that the serum level of a GLP-1 protein is similar to that of controls, but its receptor level is lower than in controls, could be explained by receptor downregulation or loss of receptor expression at the target cells, and not a problem with the protein itself. Key possible mechanisms involve ligand-induced receptor downregulation by a chronic or past exposure to higher levels of the protein (hormone, cytokine, drug), which can trigger cells to internalize and degrade the receptor, so fewer receptors remain on the surface even if the current serum protein level looks normal. This leads to decreased sensitivity despite normal circulating protein levels. Another possibility includes genetic or epigenetic changes in receptors, such as mutations or regulatory changes in the receptor gene or its promoter that can reduce receptor transcription, translation, or increase degradation, so serum protein stays normal, but cells express fewer receptors. In addition, a tissue-specific regulation could influence a receptor expression that is often regulated independently in different tissues and does not always follow serum ligand levels. Local signaling pathways and feedback loops can suppress receptor production even when circulating concentrations are unchanged. Finally, receptor obstruction or damage by autoantibodies, chronic inflammation, or toxic injury can damage receptors or promote their clearance, again lowering receptor number without necessarily changing serum ligand. Functionally, this pattern means the system is less responsive to the protein: normal blood concentration, but reduced cellular response due to fewer “docking sites” for the protein.

Combination #4 consists of curcumin, ginseng root extract, white kidney bean extract, fenugreek seed extract, and capsaicin. Existing preclinical and clinical data for these agents are broadly concordant with the present findings. It was shown that curcumin enhances GLP-1 secretion in L-cell models via Ca^2+^- and CaMKII-dependent mechanisms, with its metabolic products likely mediating GLP-1 secretagogue activity. It also modestly inhibits DPP-4, which may prolong GLP-1 action *in vivo* [36,37,38,39]. Ginsenosides, particularly Rg3, stimulate GLP-1 release through sweet-taste receptor–Gαgust-PLCβ2–IP3–Ca2^+^ signaling, and ginseng saponins improve glycemia and dyslipidemia in rodent diabetes and obesity models, partly via incretin pathways [40,41,42,43,44]. Capsaicin activates the TRPV1 receptor on sensory and enteroendocrine cells, increasing energy expenditure and GLP-1 secretion and reducing orexigenic signaling, thereby exerting both thermogenic and incretin-mediated effects [45,46,47,48]. In contrast, white kidney bean extract primarily inhibits pancreatic α-amylase, reducing carbohydrate digestion and postprandial glycemia, while fenugreek appears to act through delayed nutrient absorption, appetite modulation, and anti-inflammatory effects in adipose tissue. Neither of them has compelling evidence for direct GLP-1 pathway engagement [49,50,51,52,53,54,55,56,57,58]. Thus, within combination #4, curcumin, ginsenosides, and capsaicin likely provide the main GLP-1–related and energy-expenditure effects, whereas white kidney bean and fenugreek could contribute to upstream modulation of nutrient handling and adipose inflammation. Our study did not show significant differences in metabolic responses to composition #4 ingredients between C57BL/6 and Gulo (−/−);hLp-a+ mice strains. Although Gulo (−/−);hLp-a+ mice on HFD displayed higher total cholesterol, LDL and lower HDL serum levels compared to C57BL/6 mice on HFD, perhaps due to hLp-a insertion.

Our results line up with emerging frameworks highlighting that complex mixtures of phytochemicals rather than isolated compounds exert broader protective effects against chronic low-grade inflammation and adipocyte hypertrophy by addressing redundant metabolic networks [30,31]. A limitation of the current study is the absence of a pharmacological GLP-1 receptor agonist comparator group to benchmark the absolute magnitude of the observed effects. However, this study was designed as an initial discovery screen to evaluate the physiological feasibility of a novel oral botanical secretagogue targeting upstream, endogenous GLP-1 release. Because our mechanism focuses on stimulating natural secretion rather than introducing high-dose synthetic agonists, a direct parity comparison was outside our primary scope. Nonetheless, our observed efficacy aligns favorably when contextualized alongside published baseline data for clinical GLP-1 agonists, supporting the therapeutic potential of this endogenous approach.

While our findings corroborate established literature on the metabolic benefits of curcumin, ginseng, bitter melon, capsaicin, white kidney bean, and fenugreek, we acknowledge that the precise downstream mechanisms remain inferential. Because this initial study did not utilize GLP-1 receptor antagonists, such as Exendin(9–39), or genetic knockdown models, the causal relationship between stimulated endogenous GLP-1 and the observed systemic metabolic improvements cannot be definitively isolated. Similarly, the absolute requirement of AMPK activation remains speculative in the absence of blocking pharmacological interventions, such as dorsomorphin (Compound C). Consequently, while the parallel upregulation of these pathways strongly correlates with the observed improvements in energy expenditure and weight reduction, we cannot completely rule out the contributions of parallel, GLP-1-independent metabolic cascades. Future mechanistic studies using tissue-specific GLP-1R deletion and specific pathway inhibitors are required to confirm pathway dependence, map the exact necessity of this receptor, and fully validate these proposed cellular mechanisms.

These findings might be interpreted in the context of the current obesity treatment landscape [59]. Long-acting GLP-1 receptor agonists such as semaglutide yield mean weight reductions of approximately 15–17% in large phase 3 clinical trials when combined with lifestyle modification and represent the current pharmacologic benchmark for obesity management in people with and without diabetes [60]. By comparison, phytochemicals that modulate the GLP-1 system generally produce modest improvements in glycemia and body weight, consistent with enhancement of endogenous GLP-1 rather than pharmacologic receptor agonism [61,62,63]. Issues of bioavailability, dose, and product standardization further limit direct translation of preclinical doses to routine clinical use.

Accordingly, combination #4 and related formulations are best viewed as potential safe adjuncts to lifestyle interventions involving relevant multiple metabolic targets and, where appropriate, established pharmacotherapies. Distinguishing primary, compound-driven metabolic benefits from the secondary effects of reduced caloric intake presents a classic challenge in energy homeostasis models. Because GLP-1 receptor activation naturally drives satiety, reduced food consumption inevitably serves as a confounding variable when parsing out direct tissue-level mechanisms versus systemic weight-loss effects. Observed therapeutic improvements could be a blended outcome of direct active ingredient signaling and secondary reductions in total energy intake. Crucially, while the *in vitro* pAMPK upregulation suggests a potential baseline capacity of Combination #4 to interact with energy-sensing pathways independent of systemic feeding behavior, this cell-culture finding cannot be extrapolated to infer systemic metabolic actions *in vivo*. Because direct tissue confirmation is missing, any proposed direct upstream signaling cascade remains strictly hypothetical. Therefore, while appetite suppression and weight loss undoubtedly contribute to the phenotypic changes, the degree to which they operate alongside or downstream of an independent metabolic effect requires deep downstream validation. Additional studies testing the clinical efficacy of combination #4 in randomized trials in people with obesity are warranted to resolve potential questions and fill existing gaps.

Several further limitations of this study should be acknowledged as well. Food intake was modestly reduced in treated animals compared to animals on HFD; therefore, additional pair-feeding studies are needed to distinguish direct metabolic effects from secondary effects of reduced caloric intake. In addition, insulin sensitivity was not directly assessed using glucose or insulin tolerance testing, and mechanistic studies confirming GLP-1 pathway involvement were not performed. Also, the gut microbiota was not assessed, despite its known role as a key mediator in phytochemical metabolism. Adding pharmacokinetic studies would help bridge current gaps regarding translational efficacy, and AMPK activation was analyzed in a limited manner. Profiling the gene expression of relevant metabolic markers, alongside experiments utilizing GLP-1R antagonists and AMPK inhibitors, is necessary to establish clear causality as well. Finally, evaluating the liver, skeletal muscle, and hypothalamus is warranted, given the fundamental role these organs play in systemic metabolic regulation.

Beyond assessing pharmacokinetics, bioavailability, long-term safety, and the relative contributions of individual formulation components, future studies should follow several directions. Firstly, investigations should examine the individual bioavailability of the formulation components to identify which compounds primarily drive the observed therapeutic effects. Secondly, experiments using GLP-1 receptor antagonists and pharmacological AMPK inhibitors are required to validate in-depth the underlying mechanisms. Thirdly, transcriptomic, proteomic, and metabolomic profiling could be employed to further elucidate the modulated molecular pathways. Also, long-term studies are encouraged to evaluate metabolic and toxicological safety, alongside expanded animal models to investigate impacts on hepatic steatosis, insulin resistance, and systemic inflammation. Moreover, randomized clinical trials will be necessary to determine if these experimental model effects can be successfully reproduced in individuals with obesity. Finally, exploring hypothalamic signaling nodes (like POMC/NPY expression) and metabolic cage metrics like oxygen consumption (V_O2_) would enrich our understanding of the formulation’s central effects. While the multi-targeted mechanism of the selected ingredients is well-documented, formal mathematical synergy profiling remains an essential avenue for future translational verification as well.

## 5. Conclusions

In conclusion, our multi-component botanical formulation presents preclinical therapeutic potential to attenuate diet-induced obesity, highlighting the need for rigorous translational and clinical trials to determine human efficacy.

## Figures and Tables

**Table 1:** Summary of commercially obtained botanical extracts and phytochemical characterization based on manufacturer’s specifications.

Botanical Extract	Botanical Name	Vendor/ Product No.	Principal Marker Compounds	Major Phytochemical Class	Standardization/ Specification	Analytical Method	Analytical Reference
African mango extract	*Irvingia* *gabonensis*	Selleck Chemicals/ No. E3470	Ellagic acid derivatives, flavonoids, fatty acids	Polyphenols, lipids	Supplier specification	HPLC, 1H NMR	COA
Ginseng root extract	*Panax* *ginseng*	Sigma/ No. 05115001	Ginsenosides Rb1, Rg1, Re, Rc	Triterpenoid saponins	Total ginsenosides (%)	HPLC, 1H NMR	COA
Juniper berry extract	*Juniperus communis*	Selleck Chemicals/ Sigma No. E3172	α-Pinene, sabinene, limonene, myrcene	Monoterpenes	Volatile oil profile	GC-MS	COA
White kidney bean extract	*Phaseolus vulgaris*	Selleck Chemicals/ No. E3038	Phaseolamin (α-amylase inhibitor), lectins	Proteins, flavonoids	α-Amylase inhibition activity	Activity assay, HPLC	COA
Bitter melon extract	*Momordica charantia*	Selleck Chemicals/ No. E3396	Charantin, cucurbitane triterpenoids, momordicosides	Steroidal triterpenoids	Supplier specification	HPLC	COA
Fenugreek seed extract	*Trigonella foenum-graecum*	Selleck Chemicals/ No. E3297	Diosgenin, trigonelline, 4-hydroxyisoleucine	Steroidal saponins, alkaloids	Total saponins (%)	HPLC, IR	COA
Saffron extract	*Crocus* *sativus*	Sigma/ No. S8381	Crocin, crocetin, picrocrocin, safranal	Apocarotenoids	Crocin content (%)	HPLC, IR	COA
Ginger extract	*Zingiber* *officinale*	Sigma/ No. CH6H8C2C5042	6-gingerol, 8-gingerol, 10-gingerol, shogaols	Phenolic ketones	Total gingerols (%)	HPLC, IR	COA

**Table 2:** Test compositions used in the *in vitro* study.

Type of Composition	Formulation
Composition #1	ginseng root extract, capsaicin, hydroxycitric acid, diindolylmethane, bitter melon extract, fenugreek seed extract
Composition #2	ginseng root extract, capsaicin, hydroxycitric acid, curcumin, bitter melon extract, fenugreek seed extract
Composition #3	ginseng root extract, capsaicin, hydroxycitric acid, diindolylmethane, bitter melon extract, white kidney bean extract
Composition #4	ginseng root extract, capsaicin, curcumin, bitter melon extract, fenugreek seed extract, white kidney bean extract
Composition #5	African mango extract, capsaicin, diindolylmethane, fucoxanthin
Composition #6	African mango extract, ginseng root extract, fenugreek seed extract, capsaicin, diindolylmethane, fucoxanthin

**Table 3:** Specification of the test composition #4 used in the *in vivo* study.

Additives Included in HFD	Concentration (%)
Ginseng root extract	0.08
Capsaicin	0.03
Curcumin	0.1
White kidney bean extract	0.1
Fenugreek seed extract	0.09
Bitter melon extract	0.1

**Table 4:** Effects of test diets on body weight in C57BL/6 and Gulo (−/−);hLp-a+ mice.

	**Day 0 (g)**		**Day 77 (g)**	
**Group Study**	**C57BL/6 Mice**	**Gulo/Lp(a)** **Mice**	**C57BL/6 Mice**	**Gulo/Lp(a)** **Mice**
ND	20.2 ± 2.1	22.2 ± 3.6	25.6 ± 2.0	28.9 ± 3.6
HFD	20.5 ± 2.9	22.7 ± 2.3	41.6 ± 3.5 ^&^	40.5 ± 3.4 ^&^
HFD + A	20.6 ± 2.2	22.5 ± 3.3	32.5 ± 2.9 *^a^	31.9 ± 2.7 ^a^
**Total Energy Intake (77 days, kcal)**			**Final FER (77 days, mg/kcal)**	
	**C57BL/6 Mice**	**Gulo/Lp(a)** **Mice**	**C57BL/6 Mice**	**Gulo/Lp(a)** **Mice**
ND	740.0	692.2	7.30	9.68
HFD	1455.3	1330.6	14.50	13.38
HFD + A	1205.8	1039.5	9.87	9.04

Gulo/Lp(a) mice—Gulo (−/−);hLp-a+ strain; ND—normal diet, HFD—high-fat diet, HFD + A—high-fat diet + additives; FER—Feed Efficiency Ratio. Longitudinal data collected twice per week across 22 time points were analyzed via a Two-Way Repeated-Measures (RM) ANOVA to evaluate the main effects of treatment, time, and their interaction, followed by Tukey’s post hoc multiple comparisons test. Sphericity was evaluated via Mauchly’s test and corrected using Greenhouse–Geisser estimates. For C57BL/6 mice: treatment main effect: (F(2, 21) = 389.84, *p* < 0.001; time effect: F(21,441) = 177.10, *p* < 0.001; interaction: F(42,441) = 32.32, *p* < 0.001; for Gulo/Lp(a) mice: treatment main effect: F(2, 21) = 245.36, *p* < 0.001; time effect: F(21, 441) = 162.15, *p* < 0.001; interaction: F(42,441) = 26.84, *p* < 0.001. For terminal Day 77 pairwise body weight comparisons: & *p* < 0.001 between ND and HFD, * *p* < 0.01 between ND and HFD + A, ^a^ *p* < 0.01 between HFD and HFD + A by Tukey’s post hoc test. Data are presented as mean ± SD (*n* = 8/group).

**Table 5:** Effects of test diets on food and water in C57BL/6 and Gulo (−/−);hLp-a+ mice.

	Food (g)		Water (g)	
Group Study	C57BL/6 Mice	Gulo/Lp(a) Mice	C57BL/6 Mice	Gulo/Lp(a) Mice
ND	3.1 ± 0.51	2.9 ± 0.71	2.5 ± 0.60	2.4 ± 0.78
HFD	3.5 ± 0.69	3.2 ± 0.70	2.6 ± 0.75	2.5 ± 0.78
HFD + A	2.9 ± 0.49	2.5 ± 0.20	2.8 ± 0.43	2.6 ± 0.42

Gulo/Lp(a) mice—Gulo (−/−);hLp-a+ strain; ND—normal diet, HFD—high-fat diet, HFD + A—high-fat diet + additives. Longitudinal data collected twice per week across 22 time points were analyzed via a Two-Way Repeated-Measures (RM) ANOVA to evaluate the main effects of treatment, time, and their interaction, followed by Tukey’s post hoc multiple comparisons test. Sphericity was evaluated via Mauchly’s test and corrected using Greenhouse–Geisser estimates. For food intake profiling, the C57BL/6 metrics revealed a treatment main effect of F(2, 21) = 742.25, *p* < 0.001; time effect of F(21, 441) = 31.07, *p* < 0.001; and interaction of F(42, 441) = 25.56, *p* < 0.001. The corresponding Gulo/Lp(a) food metrics yielded a treatment main effect of F(2, 21) = 342.27, *p* < 0.001; time effect of F(21, 441) = 34.08, *p* < 0.001; and interaction of F(42, 441) = 31.52, *p* < 0.001. For water consumption profiling, the C57BL/6 strain yielded a treatment main effect of F(2, 21) = 19.87, *p* < 0.001; time effect of F(21, 441) = 9.06, *p* < 0.001; and interaction of F(42, 441) = 7.86, *p* < 0.001. The Gulo/Lp(a) strain water metrics yielded a treatment main effect of F(2, 21) = 142.61, *p* < 0.001; time effect of F(21, 441) = 11.23, *p* < 0.001; and interaction of F(42, 441) = 9.45, *p* < 0.001. Subsequent Tukey’s post hoc pairwise multiple comparisons between specific diet groups (ND vs. HFD, ND vs. HFD + A, and HFD vs. HFD + A) across chronological intervals revealed no statistically significant differences (*p* > 0.05). Data are presented as mean ± SD (*n* = 8/group).

**Table 6:** Effects of test diets on WAT in C57BL/6 and Gulo (-/-);hLp-a+ mice.

	Subcutaneous Fat (g)		Visceral Fat (g)		Mesenteric Fat (g)	
Group Study	C57BL/6 Mice	Gulo/Lp(a) Mice	C57BL/6 Mice	Gulo/Lp(a) Mice	C57BL/6 Mice	Gulo/Lp(a) Mice
ND	1.2 ± 0.03	1.5 ± 0.01	1.4 ± 0.02	1.1 ± 0.02	0.1 ± 0.001	0.2 ± 0.02
HFD	3.4 ± 0.02 **	2.9 ± 0.03 **	4.0 ± 0.01 **	5.5 ± 0.02 **	0.5 ± 0.012 **	0.8 ± 0.03 **
HFD + A	1.7 ± 0.02 ^a^	1.8 ± 0.02 ^a^	2.4 ± 0.02 *^a^	2.5 ± 0.01 *^a^	0.1 ± 0.001 ^a^	0.3 ± 0.02 ^a^

Gulo/Lp(a) mice—Gulo (−/−);hLp-a+ strain, ND—normal diet, HFD—high-fat diet, HFD + A—high-fat diet + additives. Data were analyzed within each respective genetic strain using separate, independent two-tailed Student’s *t*-tests to evaluate planned pairwise differences (ND vs. HFD, ND vs. HFD + A, and HFD vs. HFD + A) across a total of 14 degrees of freedom (*df* = 14) per individual comparison. To maintain statistical rigor and control the family-wise error rate across multiple concurrent endpoints, a Bonferroni multiple-comparison correction was applied, adjusting the critical significance threshold to α= 0.0167. * *p* < 0.01 between ND and HFD + A, ** *p* < 0.001 between ND and. HFD; ^a^ *p* < 0.001 between HFD and HFD + A. Data are presented as mean ± SD (*n* = 8/group).

**Table 7:** Effects of test diets on obesity relevant biochemical parameters in sera of C57BL/6 and Gulo (−/−);hLp-a+ mice.

	C57BL/6 Mice			Gulo/Lp(a) Mice		
Parameter	ND	HFD	HFD + A	ND	HFD	HFD + A
Cholesterol (mg/dL)	112 ± 33.6	182 ± 45.3 *	126 ± 48.1 ^a^	157 ± 43.2	264 ± 51.2 *	197 ± 49.8 ^a^
HDL (mg/dL)	55 ± 6.9	84 ± 5.8 *	87 ± 6.7 ^	40 ± 5.4	68 ± 6.4 *	64 ± 6.3 ^
LDL (mg/dL)	36 ± 5.6	54 ± 6.1 *	26 ± 6.9 ^a^	42 ± 6.2	77 ± 6.6 *	34 ± 7.9 ^a^
Triglycerides (mg/dL)	69 ± 13.5	107 ± 29.7 ^&^	72 ± 27.2 ^a^	54 ± 8.6	92 ± 9.6 *	61 ± 7.9 ^a^
Lipase (U/L)	56 ± 6.3	21 ± 4.9 *	52 ± 8.1 ^a^	57 ± 6.3	37.9 ± 1.9 *	67 ± 5.1 ^a^
Lipemia index	Normal	+	Normal	Normal	+	Normal
AST (U/L)	173 ± 12.3	182 ± 26.3	174 ± 23.3	130 ± 15.6	128 ± 22.8	135 ± 19.8
ALT (U/L)	57 ± 4.3	59 ± 5.1	57 ± 4.9	53 ± 8.8	56 ± 8.1	52 ± 7.2
GGT (U/L)	<1	<1	<1	<1	<1	<1
BUN (mg/dL)	33 ± 4.5	31 ± 3.9	32 ± 4.2	27 ± 3.9	22 ± 3.7	27 ± 5.0
Glucose (mg/dL)	106 ± 63.2	118 ± 58.1 ^#^	109 ± 59.2 ^b^	108 ± 44.3	131 ± 50.1 ^#^	106 ± 52.3 ^b^
Hemolysis index	Normal	Normal	Normal	Normal	Normal	Normal

Gulo/Lp(a) mice—Gulo (−/−);hLp-a+ strain; ND—normal diet, HFD—high-fat diet, HFD + A—high-fat diet + additives. To evaluate variations across physiological dimensions while preventing multi-endpoint Type I error rate inflation, parameters were compartmentalized into independent hypothesis families. Within each family, data were analyzed using separate, independent One-Way ANOVAs, yielding a consistent design variance profile of F(2, 21) across all parameters, followed by Tukey’s post hoc multiple comparisons test to isolate specific pairwise changes. *p*-value thresholds within families were strictly maintained as follows: # *p* < 0.05, & *p* < 0.01, * *p* < 0.001 between ND and HFD; ^a^ *p* < 0.001, ^b^ p < 0.05 between HFD and HFD + A; ^ *p* < 0.001, between ND and HFD + A. Data are presented as mean ± SD (*n* = 8/group).

## Data Availability

The data sets generated during and/or analyzed during the current study are available from the corresponding author on reasonable request.

## Supplementary Materials

The following supporting information can be downloaded at: https://www.mdpi.com/article/10.3390/nu18132111/s1, Table S1: Candidate Formulation Screening Matrix and Selection Criteria.
