# Supplementary material for: Dual Activation of GLP-1 and AMPK Pathways by a Multi-Botanical Formulation Improves Obesity and Metabolic Dysfunction in Experimental Models
Source: Nutrients. 2026 Jun 28;18(13):2111. doi: 10.3390/nu18132111 (PMC13363309; doi:10.3390/nu18132111)
Supplement: Supplementary file 1 [file nutrients-18-02111-s001.zip › nutrients-4382953-supplementary.pdf]

Table S1: Candidate Formulation Screening Matrix and Selection Criteria.

Formulation ID	GLP-1 induction (Fold Change)	Lipid accumulation reduction (%)	pAMPK level (%)	Selection status
Composition #1	High	Moderate	Low	Eliminated: Insufficient metabolic efficacy
Composition #2	Moderate	Minor	Low	Eliminated: Insufficient metabolic efficacy
Composition #3	Low	Moderate	Low	Eliminated: Insufficient GLP-1 induction
**Composition #4**	**High**	**Robust**	**Moderate**	**SELECTED: Most robust, balanced profile**
Composition #5	Low	Minor	Low	Eliminated: Insufficient GLP-1 induction and Insufficient metabolic efficacy
Composition #6	Low	Minor	Low	Eliminated: Insufficient GLP-1 induction and Insufficient metabolic efficacy
